# Resveratrol Improves the Energy Sensing and Glycolipid Metabolism of Blunt Snout Bream *Megalobrama amblycephala* Fed High-Carbohydrate Diets by Activating the AMPK–SIRT1–PGC-1α Network

**DOI:** 10.3389/fphys.2018.01258

**Published:** 2018-09-11

**Authors:** Hua-Juan Shi, Chao Xu, Ming-Yang Liu, Bing-Ke Wang, Wen-Bin Liu, Dan-Hong Chen, Li Zhang, Chen-Yuan Xu, Xiang-Fei Li

**Affiliations:** ^1^Key Laboratory of Aquatic Nutrition and Feed Science of Jiangsu Province, College of Animal Science and Technology, Nanjing Agricultural University, Nanjing, China; ^2^Wuxi Fisheries College, Nanjing Agricultural University, Wuxi, China; ^3^Key Laboratory of Freshwater Fisheries and Germplasm Resources Utilization, Ministry of Agriculture, Freshwater Fisheries Research Center, Chinese Academy of Fishery Sciences, Wuxi, China

**Keywords:** growth performance, energy sensing, glycolipid metabolism, resveratrol, blunt snout bream

## Abstract

This study investigated the effects of resveratrol on the growth performance, energy sensing, glycolipid metabolism and glucose and insulin load of blunt snout bream *Megalobrama amblycephala* fed high-carbohydrate diets. Fish (39.44 ± 0.06 g) were randomly fed three diets: a control diet (30% carbohydrate), a high-carbohydrate diet (HC, 41% carbohydrate), and the HC diet supplemented with 0.04% resveratrol (HCR) for 12 weeks. Fish fed the HC diet had significantly high values of nitrogen and energy retention efficiency, hepatosomatic index, intraperitoneal fat ratio, whole-body lipid content and intraperitoneal fat glycogen and lipid contents compared to the control group, but showed little difference with the HCR treatment. Liver and muscle lipid contents and plasma levels of glucose, glycated serum protein, advanced glycation end products and total cholesterol of fish fed the HC diet were significantly higher than those of the control group, whereas the opposite was found with resveratrol supplementation. Fish fed the HC diet obtained significantly low values of plasma insulin levels and hepatic adenosine monophosphate (AMP) contents and NAD^+^/NADH ratio compared to HCR treatment, but showed little difference with the control group. The opposite was found for hepatic adenosine triphosphate (ATP) contents and the ATP/AMP ratio. In addition, fish fed the HC diet showed significantly high transcriptions of glucose transporter 2 (GLUT2), glucose-6-phosphate dehydrogenase, glycogen synthase, fatty acid synthetase (FAS), acetyl-CoA carboxylase α (ACCα), peroxisome proliferator-activated receptor γ and PPARα compared to the control group, whereas the opposite was found for protein levels of AMP-activated protein kinase α (t-AMPKα), phosphorylated AMP-activated protein kinase α (p-AMPKα), sirtuin-1 (SIRT1), and p-AMPKα/t-AMPKα ratio as well as the transcriptions of AMPKα1, AMPKα2, SIRT1, PPARγ coactivator-1α (PGC-1α), phosphoenolpyruvate carboxykinase, fructose-1,6-bisphosphatase (FBPase), glucose-6-phosphatase, carnitine palmitoyl transferase I (CPT I) and acyl-CoA oxidase. Resveratrol supplementation significantly up-regulated the protein levels of t-AMPK, p-AMPK, and SIRT1, p-AMPK/t-AMPK ratio as well as the transcriptions of AMPKα1, AMPKα2, SIRT1, PGC-1α, GLUT2, FBPase, and CPT I compared to HC group, while the opposite was found for sterol regulatory element-binding protein-1, FAS and ACCα. Furthermore, resveratrol improved glucose and insulin tolerance of fish fed the HC diet after glucose and insulin load.

## Introduction

Carbohydrates are generally deemed to the most economical energy source in fishery, because of their relatively low cost and protein-sparing effect (namely spare protein from being catabolized for energy, and decrease the ammonia emission into aquatic environment) (Shiau and Lin, 2001; Watanabe, 2002). The inclusion of high levels of carbohydrates is favored by the aquaculture industry, as could usually result in a decrease in feed cost. However, unlike the case of most terrestrial animals, fish generally have a limited ability to use glucose for energy purposes (Polakof et al., 2012; Wang et al., 2016). In addition, the nutritional values of carbohydrates vary greatly among different fish species. Generally, the omnivorous and herbivorous fish are capable to utilize much higher levels of carbohydrates than carnivorous ones (Wilson, 1994). To date, the metabolic differences in carbohydrate utilization among fish species are not still fully understood. Recently, some studies have shown that the low utilization of carbohydrates by fish might be partly due to the poor postprandial supervision of certain energy metabolic sensors, which are closely involved in glucose metabolism (Magnoni et al., 2012; Polakof et al., 2012; Condesieira and Soengas, 2017; Kamalam et al., 2017). Indeed, some studies have shown that the intermediary metabolism of fish could be regulated by these energy sensors that control intracellular glucose use (Lu et al., 2018; Xu et al., 2018). In view of this, the molecular investigations of these energy sensors might promote our understanding of the carbohydrate utilization by fish.

The AMP-activated protein kinase (AMPK) is an evolutionary conserved serine/threonine protein kinase. As a key energy metabolic sensor, it plays a vital important role in maintaining the cellular energy homeostasis (Foretz and Viollet, 2011). Previous studies have showed that AMPK could activate the silent information regulator 1 (SIRT1), a NAD-dependent deacetylases, which is a main regulator of energy metabolism through the regulation of the transcription factors and coregulators via deacetylation (Cantó and Auwerx, 2009; Wu et al., 2011). Among them, peroxisome proliferators γ-activated receptor coactivator-1α (PGC-1α) is of vital importance, since it is a main regulator of fatty acid β-oxidation and gluconeogenesis (Cantó and Auwerx, 2009). Indeed, the AMPK–SIRT1–PGC-1α system is considered as an energy sensing network that controls the energy expenditure of animals (Cantó and Auwerx, 2009). Now, it is well acknowledged that AMPK could be activated through the increase of the AMP/ATP ratio. Once activated, AMPK could inhibit hepatic glucose output, adipogenesis and cholesterol synthesis, but stimulate glucose uptake and fatty acid oxidation accompanied by a simultaneous modulation of insulin production by strengthening the survival ability of pancreatic beta-cells (Winder and Hardie, 1999; Viollet et al., 2009; Magnoni et al., 2012). Generally, these metabolic adjustments are accomplished by the assistance of both SIRT1 and PGC-1α (Rodgers et al., 2005). This again indicated that AMPK, SIRT1 and PGC-1α play an important role in regulatory network for metabolic homeostasis. However, the aforementioned studies mainly focus on mammals (especially in rats). The potential role of this energy sensing network in the intermediary metabolism of fish is still barely understood.

Resveratrol (3,4′,5-trihydroxy-stilbene) is a naturally occurring phytoalexin. This compound is found in different pharmaceutical dosage forms, and is recommended as a dietary supplement (Lekli et al., 2008; Bhatt et al., 2012; Krithika et al., 2015). Previous studies have demonstrated that resveratrol has anti-diabetic properties by the improvement of insulin sensitivity, stimulation of glucose uptake and enhancement of lipolysis and fatty acid β-oxidation (Breen et al., 2008; Vallianou et al., 2013). Indeed, resveratrol has been demonstrated to alleviate the negative metabolic effects of excess calorie intake, improve glucose tolerance, prevent the development of fatty liver, and enhance mitochondrial biogenesis in obese rodents (Baur and Sinclair, 2006; Lagouge et al., 2006). Several studies have suggested that these metabolic actions might be due to the mediation of AMPK. In fact, resveratrol could activate AMPK, which in turn induces the expression of SIRT1. This subsequently activates the activity of PGC-1α through deacetylation, thereby stimulating the catabolic pathways and inhibiting the anabolic ones (Ajmo et al., 2008; Wu et al., 2011). This indicated that the beneficial effects of resveratrol on the glucose and lipid metabolism of animals might be accomplished through the mediation of the AMPK–SIRT1–PGC-1α network. However, these information have mainly derived in terrestrial animals. Studies regarding the beneficial effects of resveratrol on the intermediary metabolism of aquatic animals are still quite scared. To date, only three literatures are available investigating the beneficial effects of resveratrol on the lipid metabolism of zebrafish *Danio rerio*, blunt snout bream *Megalobrama amblycephala*, Atlantic salmon (*Salmo salar*) fed high-fat diets (Ran et al., 2017; Zhang et al., 2017; Menoyol et al., 2018). However, such information concerning carbohydrate metabolism is still absent. In addition, the underlying mechanisms are still poor understood, as warranted further in-depth studies.

Blunt snout bream is one of the most important economic freshwater fish in China. Due to its fast growth and high larval survival rate, this species has been widely cultured worldwide (Li et al., 2012). However, due to its herbivorous feeding habit, diets formulated for this species usually contain large amounts of carbohydrates, as inevitably results in severe metabolic disorders (Prathomya et al., 2017; Prisingkorn et al., 2017). Therefore, investigating the carbohydrate utilization by this species and the underlying mechanisms are extremely important. Considering this, the present study was conducted to evaluate the long-term influences of dietary resveratrol supplementation on the growth performance, energy sensing, glycolipid metabolism and glucose and insulin load of this fish fed carbohydrate-enriched diets. The results presented here could provide us some new insights into the carbohydrate metabolism of fish, as might facilitate the use of resveratrol in the aquaculture industry.

## Materials and Methods

### Ethics Statement

All experimental animals in the present study were approved by the Animal Care and Use Committee of Nanjing Agricultural University (Nanjing, China). All experimental animal operations were approved and guided by the Care and Use of Laboratory Animals in China.

### Experimental Diets

Four isonitrogenous (32% crude protein) and isolipidic (5% crude lipid) diets were formulated, including a control diet (containing 30% nitrogen-free extract), a high-carbohydrate diet (HC, containing 41% nitrogen-free extract) and a HC diet supplemented with 0.04% resveratrol (HCR). Dietary carbohydrate levels were adopted on the basis of our previous studies (Li et al., 2013, 2014). Resveratrol used in this study was obtained from Hubei Jusheng Technology Co., Ltd., (Hubei, China), with a purity of at least 98%. The dose of resveratrol was adopted on the basis of a previous study (Zhang et al., 2017), which investigated the effects of dietary resveratrol levels on the growth and lipid metabolism of blunt snout bream fed high-lipid diets. Feed formulation and proximate composition of the experimental diets were presented in **Table 1**. Protein was supplied by fish meal, soybean meal, rapeseed meal and cottonseed meal. Dietary lipids derived from fish oil and soybean oil. Corn starch was adopted to obtain the dietary carbohydrate levels required. Microcrystalline cellulose was included as the filler.

**Table 1:** Formulation and proximate composition of the experimental diets.

Ingredients	Control	HC	HCR
Fish meal	8.00	8.00	8.00
Soybean meal	26.00	26.00	26.00
Rapeseed meal	17.00	17.00	17.00
Cottonseed meal	17.00	17.00	17.00
Fish oil	2.00	2.00	2.00
Soybean oil	2.00	2.00	2.00
Corn starch	12.00	25.00	25.00
Microcrystalline cellulose	13.00	0.00	0.00
Resveratrol (%)	0.00	0.00	0.04
Calcium biphosphate	1.80	1.80	1.80
Premix^1^	1.20	1.20	1.20
*Proximate composition (% air-dry basis)*			
Moisture	9.32	9.37	9.57
Crude protein	32.20	32.73	32.71
Crude lipid	5.58	5.38	5.71
Ash	7.05	7.07	7.12
Crude fiber	15.99	3.81	3.54
Nitrogen-free extract^2^	29.86	41.64	41.35
Energy (MJ/kg)	19.71	19.72	19.20

*Control, diet with 30% carbohydrate level; HC, diet with 41% carbohydrate level; HCR, diet with 41% carbohydrate and 0.04% resveratrol. ^1^Premix supplied the following minerals and vitamins (per kg): CuSO_4_⋅5H_2_O, 2.0 g; FeSO_4_⋅7H_2_O, 25 g; ZnSO_4_⋅7H_2_O, 22 g; MnSO_4_⋅4H_2_O, 7 g; Na_2_SeO_3_, 0.04g; KI, 0.026 g; CoCl_2_⋅6H_2_O, 0.1 g; Vitamin A, 900,000 IU; Vitamin D, 200,000 IU; Vitamin E, 4500 mg; Vitamin K_3_, 220 mg; Vitamin B_1_, 320 mg; Vitamin B_2_, 1090 mg; Vitamin B_5_, 2000 mg; Vitamin B_6_, 500 mg; Vitamin B_12_, 1.6 mg; Vitamin C, 5000 mg; pantothenate, 1,000 mg; folic acid, 165 mg; choline, 60,000 mg. ^*2*^Calculated by difference (100 – moisture – crude protein – crude lipid – ash – crude fiber).*

The diets were produced in our laboratory. Ingredients were finely ground, carefully weighed, well mixed, and pelletized using a laboratory pellet machine (MUZL 180, Jiangsu Muyang Group Co., Ltd., Yangzhou, China). All diets were dried at 30°C for 24 h. After drying, the diets were stored at −20°C in plastic bags until use.

### Experimental Fish and Feeding Trial

Blunt snout bream were purchased from the National Fish Hatchery Station in Yangzhou (Jiangsu province, China). Before the experiment, fish were acclimated to the experimental conditions by feeding a commercial diet (feed No. 191, Tongwei feed group Co., Ltd., Wuxi, China) containing 32% protein and 5% lipid for 2 weeks. After that, a total of 216 fish (average weight: 39.44 ± 0.06 g) were randomly distributed into 12 tanks (300 L each) in a flow-through recirculating aquaculture system (water flow rate, 2 L/min) at a stocking density of 18 fish per tank. Then, fish were fed to visual satiation thrice daily (07:30, 11:30, and 16:30 h) for 12 weeks with one of three experimental diets. Each diet was tested in four tanks. Throughout the feeding period, a 12:12 h light: dark regime (07:00 to 19:00 h light period) was maintained by timed fluorescent lighting. Water temperature was maintained at 27 ± 1°C, and dissolved oxygen was maintained above 5.0 mg/L. Total ammonia nitrogen and nitrite were maintained below 0.2 and 0.01 mg/L, respectively.

### Sample Collection

Before the feeding trial, six fish were randomly collected from the acclimated fish for the analysis of initial body composition. Then, the rest of fish were assigned to the feeding trial. After the last meal, fish were fasted for 24 h to empty gut contents prior to sampling. Then, all fish in each tank were counted and weighed. Four fish from each replicate with a total of 16 fish from each treatment were randomly selected, and were slightly anesthetized by MS-222 (tricaine methanesulfonate, Sigma, United States) at a concentration of 100 mg/L. Thereafter, blood sample was rapidly taken from the caudal vein using heparinized syringes, and was centrifuged at 3000 rpm at 4°C for 10 min. The supernatant was collected and stored at −80°C for subsequent analysis. It should be mentioned here that fish among different treatments were sampled at equally timed intervals (about 20 min for each replicate) to average diurnal fluctuations in hormone titres across the groups. Then, individual liver, muscle and intraperitoneal fat were also sampled. These samples were stored in liquid nitrogen for further analysis. In addition, two fish were randomly collected from each tank, and were stored at −20°C for the determination of whole-body composition.

### Growth Performance Determination

The growth performance parameters adopted in this study were calculated as follows:

Feed intake (g per fish)=total feed intake (g)=total fish number.

$$\mathrm{Weight gain rate}\;(\text{WGR},\%)=[(W_{\text{t}}-W_{0})/W_{0})]*100.$$

$$\mathrm{Specific growth rate}\;(\text{SGR},\;\%)=[(LnW_{\text{t}}-\text{Ln}W_{0})/T]*100.$$

Feed conversion ratio (FCR)=total feed intake (g)=total weight gain (g).

Protein efficiency ratio (PER)=fish weight gain (g)=total protein fed (g).

Hepatosomatic index (HSI, %)=(liver weight/body weight)*100.

Viscera index (VSI, %)=(viscera weight/body weight)*100.

Intraperitoneal fat ratio (IPF, %)=(intraperitoneal fat weight/body weight)*100.

$$\mathrm{Retention of nitrogen}\;(\text{NRE},\;\%)=((W_{\text{t}}^{*}N_{\text{t}}-W_{0}^{*}N_{0})/(N_{\text{diet}}*\;\mathrm{feed intake})*100.$$

$$\mathrm{Retention of energy}\;(\text{ERE},\;\%)=(W_{\text{t}}^{*}E_{\text{t}}-W_{0}^{*}E_{0})/(E_{\text{diet}}*\;\mathrm{feed intake})*100.$$

The *W* is body weight, *W*_0_ is initial weight, *W*_t_ is final weight, *T* is the culture period in days, *N*_0_/*E*_0_ and *N*_t_/*E*_t_ are the initial and final nitrogen/energy contents in whole body, respectively, and *N*_diet_/*E*_diet_ are the nitrogen/energy contents in the diets.

### Glucose Tolerance Test (GTT) and Insulin Tolerance Test (ITT)

After initial sampling, the remaining fish in each group were assembled in one tank, and were starved for 24 h before the GTT and ITT.

For GTT, according to the method detailed by Li et al. (2016), 20 fish from each treatment were slightly anesthetized and weighed. Then they received an intraperitoneal injection of glucose [1.67 g glucose per kg body weight (BW)] within 10 min to minimize the deviation of plasma glucose levels. A saline solution (0.9%) containing 100 mg glucose per mL was used for that purpose. Then, fish were immediately transferred to five tanks at a rate of four fish per tank, and were sampled at 1, 2, 4, 8, and 12 h, respectively. One tank of fish was sampled for each sampling time in order to minimize the stress due to sampling. Additionally, the blood samples collected before the GTT were used for time 0 h. Blood samples were taken following the procedures aforementioned.

The ITT was carried out as described in our previous study (Shi et al., 2018). Briefly, another 20 fish from each treatment were individually weighted and injected intraperitoneally with bovine insulin (0.052 mg/kg BW, Sigma, United States) (Jin et al., 2014) within 10 min. A saline solution (0.9%) containing either 0.052 mg insulin per mL was used for that study. Then, fish were immediately transferred to five tanks at a rate of four fish per tank, and were sampled at 1, 2, 4, 8, and 12 h, respectively, after injection following the procedures detailed in GTT.

### Analysis of Proximate Composition, Tissue Glycogen Synthase (GS) Activities, and Tissue Glycogen and Lipid Contents

Diets and fish were analyzed for proximate composition. Moisture was determined by oven drying at 105°C until constant weight. Crude protein (nitrogen × 6.25) was determined by the micro-Kjeldahl method using an Auto Kjeldahl System (FOSS KT260, Switzerland). Crude lipid was determined via ether extraction using a Soxtec System (Soxtec System HT6, Tecator, Sweden). Ash content was analyzed by burning at 550°C for 4 h. Gross energy was measured using an adiabatic bomb calorimeter (PARR 1281, Parr Instrument Company, Moline, IL, United States). Crude fiber was analyzed by fritted glass crucible method using an automatic analyzer (ANKOM A2000i, Macedon, New York, NY, United States). In addition, the crude protein, crude lipid, ash and gross energy in whole body were measured on the air-dry basis samples, which were converted to wet-weight basis samples. Tissue GS activities were determined following the procedures detailed by Villa-Moruzzi et al. (1979). Tissue glycogen and lipid contents were measured according to Folch et al. (1957) and Keppler et al. (1974), respectively.

### Analysis of Plasma and Liver Metabolites

The levels of plasma glucose was measured using the glucose oxidase method (Asadi et al., 2009). Plasma glycated serum protein (GSP) and AGES levels were assayed by the method detailed by David and John (1984) and Monnier et al. (1986), respectively. Plasma insulin level was analyzed using a heterologous radioimmunoassay method (Gutierrez et al., 1984). This method has been verified in common carp (*Cyprinus carpio* L.) (Hertz et al., 1989), which shares the same classification (the Cyprinidae family) with blunt snout bream. Plasma triglyceride and total cholesterol levels were determined by the colorimetric enzymatic methods (McNamara and Schaefer, 1987). Hepatic contents of adenosine triphosphate (ATP) and adenosine monophosphate (AMP) were measured following the procedures detailed by Adam (1965) and Lund et al. (1975), respectively. The contents of nicotinamide adenine dinucleotide (NAD^+^) and nicotinamide adenine dinucleotide phosphate (NADH) were determined by Woodley and Gupta (1971).

### Western Blot and Quantitative Real-Time PCR (RT-PCR)

Total protein was extracted from the liver using RIPA lysis buffer (#9806, Cell Signaling, Danvers, MA, United States) containing 20 mM Tris-HCl (pH 7.5), 150 mM NaCl, 1 mM Na2EDTA, 1 mM EGTA, 1% NP-40, 1% sodium deoxycholate, 2.5 mM sodium pyrophosphate, 1 mM beta-glycerophosphate, 1 mM Na3VO4, 1 μg/mL leupeptin, 1 mM PMSF and 1× protease inhibitor cocktail (Cell Signaling, Danvers, MA, United States, #5871). Then, it was centrifuged at 12,000 × *g* at 4°C for 15 min. The supernatant was collected and stored at −80°C for subsequent analysis. Then, the protein concentration was determined using a Bio-Rad Protein Assay Kit (Bio-Rad Laboratories, Munich, Germany). Subsequently, proteins were separated by sodium dodecyl sulfate-polyacrylamide gel electrophoresis (SDS-PAGE) using a Mini-Protean system (Bio-Rad, Spain) for 1–2 h at 100 V, then they were transferred to polyvinylidene fluoride (PVDF) membranes (Millipore, Danvers, MA, United States). The specific primary antibodies used were anti-β-actin (BM3873, Boster, China, 1:5000 dilution), anti-AMPKα (#2532, Cell Signaling Technology, United States, 1:2000 dilution), anti-phospho-AMPKα (#2535, Cell Signaling Technology, United States, 1:2000 dilution) and anti-SIRT1 (13161-1-AP, Proteintech, United States, 1:1000 dilution). Then PVDF membranes were washed and incubated with anti-rabbit (#7074, Cell Signaling Technology, United States, 1:2000 dilution) secondary antibody for 1–2 h at room temperature. Immune complexes were detected by a chemiluminescent substrate (Gel Imagine CHEMI-SMART-3126, France) based on the manufacturer’s instructions, and were visualized with a luminescent image analyzer (Fujifilm LAS-3000, Japan). The protein levels were normalized by β-actin, and the intensities of each lane were quantified using the densitometry band analysis tool in Image J 1.44p (United States National Institutes of Health, Bethesda, MD, United States).

Total RNA in hepatopancreas of blunt snout bream was extracted using Trizol (Invitrogen, Carlsbad, CA, United States) according to the manufacturer’s instructions, and was treated with RQ1 RNase-free DNase (Takara Co. Ltd., Japan) to eliminate genomic DNA amplification. The quantity and purity of extracted RNA were determined by absorbance measures at 260 and 280 nm, respectively. The ratio of absorbance at 260 and 280 nm is used to assess the purity of DNA and RNA. Accordingly, a ratio near 2.0 is generally accepted as “pure” for RNA. Its integrity was further measured by electrophoresis in 1.0% formaldehyde denaturing agarose gels.

cDNA was generated using 500 ng DNase-treated RNA by a RT-PCR kit (Takara Co. Ltd., Japan) following the manufacturer’s instructions. The reaction volume was 10 μL, containing 2 μL buffer (5×), 0.5 μL dNTP mixture (10 mM each), 0.25 μL RNase inhibitor (40 U μL^−1^), 0.5 μL dT-AP primer (50 mM), 0.25 μL ExScript^TM^ RTase (200 U μL^−1^) and 6.5 μL DEPC water. Cycling conditions were 42°C for 40 min, 90°C for 2 min, and 4°C thereafter.

After reverse transcription, real-time PCR was employed to determine the mRNA levels based on the SYBR Green II Fluorescence Kit (Takara Bio. Inc., Japan). The PCR primers sets were designed using the Primer 5.0 software according to the available sequences of blunt snout bream (**Table 2**). RT-PCRs were carried out on the Mini Option real-time detector (Bio-Rad, United States). The assays were performed with a reaction mixture of 20 μL per sample, each of which contained 2 μL cDNA template (equivalent to 100 ng cDNA), 0.4 μL of each primer (10 μmol L^−1^), 10 μL SYBR^®^ premix Ex Taq^TM^ (TaKaRa), 6.8 μL dH_2_O and 0.4 μL ROX Reference DyeII (TaKaRa). The PCR reaction was piloted under the following conditions: initial denaturation at 95°C for 5 s followed by 40 cycles, annealing at 60°C for 34 s and a final extension at 95°C for 5 s, followed by a melt curve analysis of 15 s from 95 to 60°C, 1 m for 60°C and then up to 95°C for 15 s. To analyze the relative transcriptional levels, the transcriptions of target genes were normalized by a reference gene-elongation factor 1 alpha (EF1α) (Zhang et al., 2013) using the 2^−ΔΔCT^ method (Livak and Schmittgen, 2001). Four samples were analyzed from each tank. It should be mentioned that all the PCRs were highly specific and reproducible (0.998 > *R*^2^ > 0.983), and all primer pairs had equivalent PCR efficiencies (from 0.89 to 1.14).

**Table 2:** Nucleotide sequences of the primers used to assay gene expressions by real-time PCR.

Target gene	Forward primer (5′–3′)	Reverse primer (5′–3′)	Accession numbers or references
AMPKα1	AGTTGGACGAGAAGGAG	AGGGCATACAAAATCAC	KX061840.1
AMPKα2	ACAGCCCTAAGGCACGATG	TGGGTCGGGTAGTGTTGAG	KX061841.1
SIRT1	TCGGTTCATTCAGCAGCACA	ATGATGATCTGCCACAGCGT	Gao et al., 2012
PGC1-α	AAGGCATAAGGGTAATCGTA	GAACGAGCTGCACTTTTCCC	Gao et al., 2012
GLUT 2	ACGCACCCGATGTGAAAGT	TTGGACAGCAGCATTGATT	KC513421.1
GK	AAAATGCTGCCCACTTAT	AATGCCCTTATCCAAATC	KJ141202.1
PK	GCCGAGAAAGTCTTCATCGCACAG	CGTCCAGAACCGCATTAGCCAC	Gao et al., 2012
PEPCK	TCGCCTGGATGAAGTTCGAC	GTCTTGGTGGAGGTTCCTGG	Gao et al., 2012
G6Pase	TTCAGTGTCACGCTGTTCCT	TCTGGACTGACGCACCATTT	Gao et al., 2012
FBPase	TACCCAGATGTCACAGAAT	CACTCATACAACAGCCTCA	KJ743995.1
GS	CCTCCAGTAACAACTCACAACA	CAGATAGATTGGTGGTTACGC	Gao et al., 2012
G6PDH	AGGTAAAGGTGCTGAAGT	AAATGTAGCCTGAGTGGA	KJ743994.1
SREBP1	GCTGGCGTGTCGCTATCT	TGTTGGCAGTCGTGGAGG	Qian et al., 2015
FAS	AGCGAGTACGGTGATGGT	GGATGATGCCTGAGATGG	KF918747.1
ACCα	TCTGCCCTCTATCTGTCT	ATGCCAATCTCATTTCCT	Qian et al., 2015
PPARγ	AGCTTCAAGCGAATGGTTCTG	AGGCCTCGGGCTTCCA	HM140627
PPARα	GTGCCAATACTGTCGCTTTCAG	CCGCCTTTAACCTCAGCTTCT	HM140628
CPT I	TACTTCCAAAGCGGTGAG	AGAGGTATTGTCCGAGCC	Lu et al., 2014
ACO	GCTCAACCCTGGCATACT	CTGGCTCAGCTTTACACG	Lu et al., 2014
EF1α	CTTCTCAGGCTGACTGTGC	CCGCTAGCATTACCCTCC	X77689.1

*AMPKα1, AMP-activated protein kinase α1; AMPKα2, AMP-activated protein kinase α2; SIRT1, sirtuin-1; PGC1-α, peroxisome proliferators γ-activated receptor coactivator-1α; GLUT 2, glucose transporter 2; GK, glucokinase; PK, pyruvate kinase; PEPCK, phosphoenolpyruvate carboxykinase; G6Pase, glucose-6-phosphatase; FBPase, fructose-1,6-biphosphatase; GS, glycogen synthase; G6PDH, glucose-6-phosphate dehydrogenase; SREBP1, sterol regulatory element-binding protein-1; FAS, fatty acid synthetase; ACCα, acetyl-CoA carboxylase α; PPARγ, peroxisome proliferator-activated receptor γ; PPARα, peroxisome proliferator-activated receptor α, CPT I, carnitine palmitoyl transferase I; ACO, acyl-CoA oxidase; EF1α, elongation factor 1 alpha.*

### Statistical Analysis

Data on growth performance, body composition, tissue GS activities and glycogen and lipid contents, plasma parameters as well as protein and gene expressions were subjected to one-way ANOVA using the SPSS 20.0 software package (SPSS Inc., Michigan Avenue, Chicago, IL, United States) for Windows, after testing the homogeneity of variances with the Levene test. Unlikely, the data regarding plasma glucose levels in the GTT and ITT were analyzed by two-way ANOVA for significant differences among treatment means based on sampling time, dietary treatments and their interaction. If significant (*P* < 0.05) differences were found in the interaction, each factor was further analyzed separately by one-way ANOVA. All data were reported as mean ± SEM (standard error of the mean).

## Results

### Growth Performance, Feed Utilization, and Whole-Body Composition

Growth performance, feed utilization and whole-body composition of blunt snout bream were presented in **Table 3**. No mortality was observed in all groups during the 12-week feeding trial. Final weight, feed intake, WGR, SGR, FCR, PER, VSI and whole-body moisture, protein, ash and energy contents showed no significant difference (*P* > 0.05) among dietary treatments. The NRE, ERE, HSI, IPF and whole-body lipid contents of fish fed the HC diet were significantly (*P* < 0.05) higher than those of the control group. But they showed no statistical difference (*P* > 0.05) with those of the HCR treatment.

**Table 3:** Growth performance, feed utilization, and whole-body composition of blunt snout bream fed different experimental diets.

Parameters	Control	HC	HCR
Initial weight (g)	39.38 ± 0.17	39.45 ± 0.17	39.53 ± 0.05
Final weight (g)	85.05 ± 2.25	89.43 ± 4.39	88.81 ± 2.64
Feed intake (g per fish)	115.00 ± 5.47	121.94 ± 6.10	118.66 ± 8.74
WGR (%)	116.10 ± 5.21	126.76 ± 2.31	124.85 ± 6.90
SGR (% day^−1^)	1.10 ± 0.03	1.16 ± 0.07	1.15 ± 0.04
FCR	2.10 ± 0.06	2.03 ± 0.09	2.09 ± 0.05
PER	1.41 ± 0.05	1.53 ± 0.05	1.51 ± 0.06
NRE (%)	22.27 ± 0.87^b^	28.34 ± 0.76^a^	28.16 ± 0.17^a^
ERE (%)	21.83 ± 0.50^b^	25.05 ± 0.66^a^	25.00 ± 0.28^a^
HSI (%)	1.13 ± 0.02^b^	1.39 ± 0.04^a^	1.31 ± 0.05^a^
VSI (%)	6.62 ± 0.27	6.47 ± 0.45	6.43 ± 0.22
IPF (%)	1.39 ± 0.07^b^	1.76 ± 0.08^a^	1.66 ± 0.06^a^
*Whole-body composition*			
Moisture (%)	70.71 ± 0.10	70.48 ± 0.67	70.78 ± 0.29
Crude protein (%)	16.80 ± 0.11	17.21 ± 0.25	17.20 ± 0.40
Crude lipid (%)	7.53 ± 0.26^b^	8.32 ± 0.12^a^	8.06 ± 0.11^ab^
Ash (%)	3.11 ± 0.04	3.49 ± 0.08	3.31 ± 0.14
Energy (MJ/kg)	7.58 ± 0.28	7.81 ± 0.16	7.86 ± 0.12

*Control, diet with 30% carbohydrate level; HC, diet with 41% carbohydrate level; HCR, diet with 41% carbohydrate and 0.04% resveratrol. Values are means ± SEM of four replications. Means in the same line with different letters were significantly different (*P* < 0.05).*

### Tissue GS Activities and Tissue Glycogen and Lipid Contents

As was shown in **Table 4**, intraperitoneal fat GS activities and muscle glycogen contents showed no significant difference (*P* > 0.05) among dietary treatments. Intraperitoneal fat lipid and glycogen contents of fish fed the HC diet were significantly (*P* < 0.05) higher than those of the control group. But, they showed no significant difference (*P* > 0.05) with those of the HCR group. In addition, liver and muscle lipid contents of fish fed the HC diet were significantly (*P* < 0.05) higher than those of the other groups. However, the HCR group obtained a significantly (*P* < 0.05) high value of liver glycogen than the other treatments.

**Table 4:** Tissue glycogen synthase activities and glycogen and lipid contents of blunt snout bream fed different experimental diets.

Parameters	Control	HC	HCR
*GS synthase activities (U/g prot)*			
Liver	20.08 ± 0.49^b^	21.50 ± 0.91^ab^	24.42 ± 0.84^a^
Muscle	17.7 ± 0.42^b^	20.52 ± 1.11^ab^	22.28 ± 0.53^a^
Intraperitoneal fat	8.71 ± 0.31	8.87 ± 0.39	9.46 ± 0.45
*Glycogen contents (mg/g)*			
Liver	6.46 ± 0.33^c^	15.20 ± 0.19^b^	18.80 ± 0.68^a^
Muscle	0.95 ± 0.02	1.06 ± 0.01	1.08 ± 0.04
Intraperitoneal fat	1.55 ± 0.01^b^	1.85 ± 0.07^a^	1.88 ± 0.05^a^
*Lipid contents (%)*			
Liver	16.95 ± 0.37^b^	20.75 ± 1.00^a^	17.07 ± 0.65^b^
Muscle	4.64 ± 0.13^b^	6.92 ± 0.30^a^	4.65 ± 0.35^b^
Intraperitoneal fat	51.28 ± 1.08^b^	60.00 ± 1.61^a^	55.80 ± 2.63^ab^

*Control, diet with 30% carbohydrate level; HC, diet with 41% carbohydrate level; HCR, diet with 41% carbohydrate and 0.04% resveratrol. Values are means ± SEM of four replications. Means in the same line with different letters were significantly different (P < 0.05).*

### Liver and Plasma Biochemistry Parameters

**FIGURE 1:**
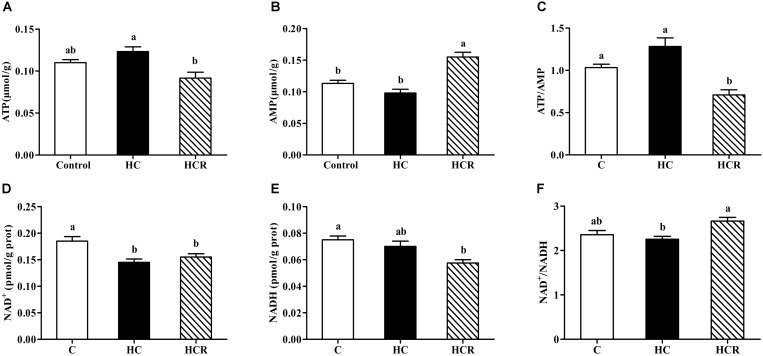
Liver ATP contents **(A)**, AMP contents **(B)**, the ATP/AMP ratio **(C)**, NAD^+^ contents **(D)**, NADH **(E)** contents and the NAD^+^/NADH ratio **(F)** of blunt snout bream fed different experimental diets. Each data represents the mean of four replicates. Bars assigned with different letters are significantly different (*P* < 0.05). Control, diet with 30% carbohydrate level; HC, diet with 41% carbohydrate level; HCR, diet with 41% carbohydrate and 0.04% resveratrol. ATP, adenosine triphosphate; AMP, adenosine monophosphate; NAD^+^, nicotinamide adenine dinucleotide; NADH, nicotinamide adenine dinucleotide phosphate.

As was shown in **Figure 1**, hepatic ATP, AMP and NADH contents as well as the ATP/AMP and NAD^+^/NADH ratios of fish fed the HC diet showed no statistical difference (*P* > 0.05) with those of the control group. The NAD^+^ content in fish fed the HC diet was significantly (*P* < 0.05) lower than that of the control group. In addition, the ATP content and the ATP/AMP ratio of the HC group were significantly (*P* < 0.05) higher than those of the HCR treatment, whereas the opposite was found for AMP content and NAD^+^/NADH ratio.

Plasma metabolites of blunt snout bream were shown in **Table 5**. No statistical difference (*P* > 0.05) was found in triglyceride levels among dietary treatments. Plasma levels of glucose, GSP and total cholesterol in fish fed the HC diet were significantly (*P* < 0.05) higher than those of the other groups. Plasma insulin levels of the HCR group was significantly (*P* < 0.05) higher than that of the other treatments. In addition, fish fed the HC diet obtained significantly (*P* < 0.05) high plasma AGES levels compared to the control group, but HC and HCR showed similar results (no significant difference).

**Table 5:** Plasma metabolites of blunt snout bream fed the different experimental diets.

Parameters	Control	HC	HCR
Glucose (mmol/L)	5.16 ± 0.35^b^	7.31 ± 0.12^a^	5.53 ± 0.20^b^
GSP (mmol/L)	1.17 ± 0.09^b^	1.64 ± 0.09^a^	1.20 ± 0.08^b^
AGES (ng/mL)	5.78 ± 0.11^b^	6.24 ± 0.09^a^	5.99 ± 0.07^ab^
Insulin (μIU/mL)	12.62 ± 0.27^b^	13.08 ± 0.33^b^	14.50 ± 0.70^a^
Triglyceride (mmol/L)	1.81 ± 0.04	1.87 ± 0.03	1.82 ± 0.02
Total cholesterol (mmol/L)	5.48 ± 0.15^b^	6.70 ± 0.15^a^	5.89 ± 0.23^b^

*Control, diet with 30% carbohydrate level; HC, diet with 41% carbohydrate level; HCR, diet with 41% carbohydrate and 0.04% resveratrol. GSP, glycated serum protein; AGES, advanced glycation end products. Values are means ± SEM of four replications. Means in the same line with different letters were significantly different (P < 0.05).*

### Hepatic Protein Expressions of AMPKα and SIRT1

**FIGURE 2:**
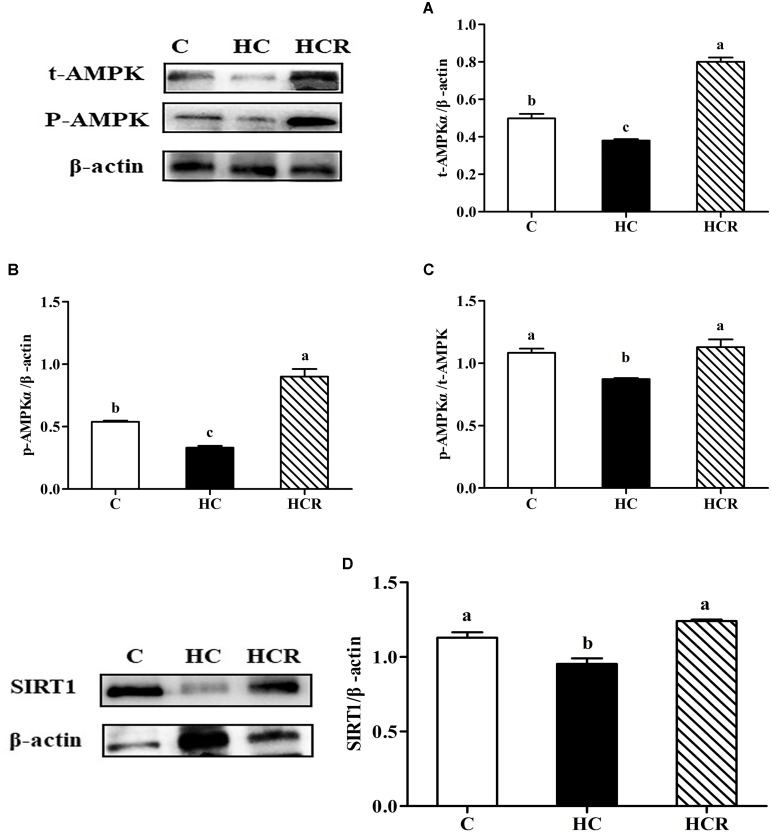
Hepatic t-AMPKα contents **(A)**, p-AMPKα contents **(B)**, the pAMPKα/t-AMPKα ratio **(C)** and SIRT1 contents **(D)** of blunt snout bream liver fed different experimental diets. Gels were loaded with 20 μg total protein per lane. Protein and phosphorylation levels were normalized to liver β-actin levels. Each data represents the mean of four replicates. Bars assigned with different letters are significantly different (*P* < 0.05). Control, diet with 30% carbohydrate level; HC, diet with 41% carbohydrate level; HCR, diet with 41% carbohydrate and 0.04% resveratrol. t-AMPKα, AMP-activated protein kinase α; phosphorylated AMP-activated protein kinase α; SIRT1, sirtuin-1.

As can be seen from **Figure 2** and **Supplementary Figure S1**, fish fed the HC diet obtained significantly (*P* < 0.05) lower t-AMPKα, p-AMPKα, the p-AMPKα/t-AMPKα, and SIRT1 protein contents compared with other groups.

### The mRNA Levels of Hepatic Enzymes Involved in Energy Sensing and Glycolipid Metabolism

**FIGURE 3:**
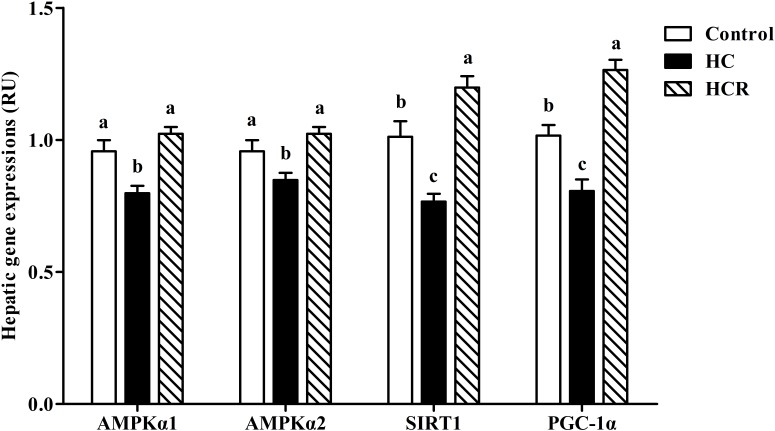
Relative expressions of the genes involved in energy sensing in the liver of blunt snout bream fed different experimental diets. Data are referred to the values (relative units, RU) obtained in fish fed the control diet. Values were normalized with the transcription of EF1α. Each data represents the mean of four replicates. Bars assigned with different letters are significantly different (*P* < 0.05). Control, diet with 30% carbohydrate level; HC, diet with 41% carbohydrate level; HCR, diet with 41% carbohydrate and 0.04% resveratrol.

As can be seen from **Figure 3**, the transcriptions of AMPKα1, AMPKα2, SIRT1, and PGC-1α of fish fed the HC diet were significantly (*P* < 0.05) lower than those of the other groups.

**FIGURE 4:**
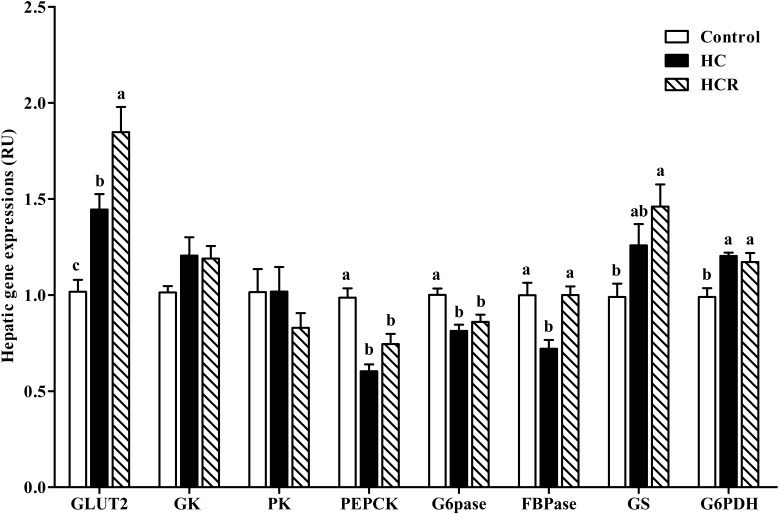
Relative expressions of the genes involved in glucose metabolism in the liver of blunt snout bream fed different experimental diets. Data are referred to the values (relative units, RU) obtained in fish fed the control diet. Values were normalized with the transcription of EF1α. Each data represents the mean of four replicates. Bars assigned with different letters are significantly different (*P* < 0.05). Control, diet with 30% carbohydrate level; HC, diet with 41% carbohydrate level; HCR, diet with 41% carbohydrate and 0.04% resveratrol.

The transcriptions of the enzymes involved in glucose metabolism were presented in **Figure 4**. Hepatic GK and PK expressions showed no significant difference (*P* > 0.05) among dietary treatments. The GLUT2 and G6PDH expressions of the control group were significantly (*P* < 0.05) lower than those of the other groups, whereas the opposite was found for PEPCK and G6Pase expressions. The GS transcription of the control group was significantly (*P* < 0.05) lower than that of the HCR treatment, but showed no statistical difference (*P* > 0.05) with that of the HC group. In addition, the FBPase expression of fish fed the HC diet was significantly (*P* < 0.05) lower than that of the other groups.

**FIGURE 5:**
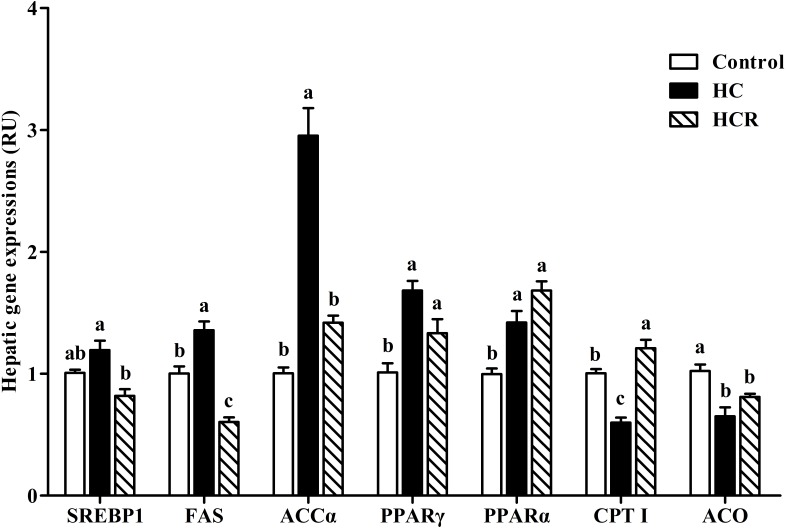
Relative expressions of the genes involved in lipid metabolism in the liver of blunt snout bream fed different experimental diets. Data are referred to the values (relative units, RU) obtained in fish fed the control diet. Values were normalized with the transcription of EF1α. Each data represents the mean of four replicates. Bars assigned with different letters are significantly different (*P* < 0.05). Control, diet with 30% carbohydrate level; HC, diet with 41% carbohydrate level; HCR, diet with 41% carbohydrate and 0.04% resveratrol.

In the **Figure 5**, the hepatic expressions of FAS and ACCα of the HC treatment were significantly (*P* < 0.05) higher than those of the other groups, whereas the opposite was found for CPT I expression. The mRNA expressions of PPARα and PPARγ in the HC group were significantly (*P* < 0.05) higher than those of the control group, but showed no statistical difference (*P* > 0.05) with that of the HCR group; whereas, the opposite was found for ACO expression. In addition, the SREBP1 expression of fish fed the HC diet was significantly (*P* < 0.05) higher than that of the HCR treatment, but HC and control group showed similar results (no significantly difference).

### Plasma Glucose Levels After the GTT and ITT

**FIGURE 6:**
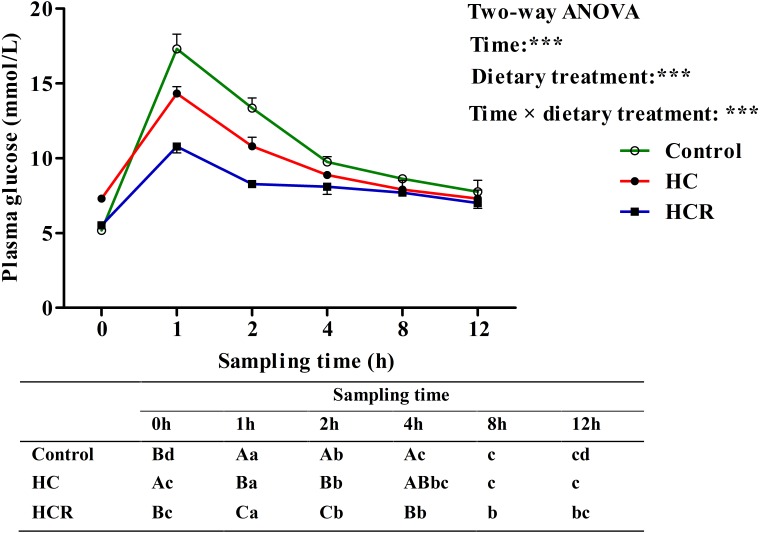
Plasma glucose levels of blunt snout bream subjected to a glucose load after the adaption to different experimental diets. Each data represents the mean of four replicates. Different lower-case letters indicate significant differences (*P* < 0.05) at different time points within each treatment, whereas different capital letters indicate significant differences (*P* < 0.05) among these three treatment at each sampling point. ^∗∗∗^*P* < 0.001. Control, diet with 30% carbohydrate level; HC, diet with 41% carbohydrate level; HCR, diet with 41% carbohydrate and 0.04% resveratrol.

As was shown in **Figure 6**, plasma glucose levels were significantly (*P* < 0.001) affected by sampling time, dietary treatments and their interaction. The glucose injecting resulted in a significantly (*P* < 0.05) elevated plasma glucose concentration with the highest value being obtained at 1 h after injection. Thereafter, plasma glucose levels decreased significantly (*P* < 0.05) to the basal value, which was attained within 8 h after the glucose load. Then gradually reduced to the minimum at 12 h. In terms of dietary treatments, plasma glucose level of the HCR treatment was significantly (*P* < 0.001) lower than that of the other groups. In addition, plasma glucose levels were significantly affected by the interaction between sampling time and dietary treatments with significant (*P* < 0.001) differences observed during the period of 0–4 h among different groups.

**FIGURE 7:**
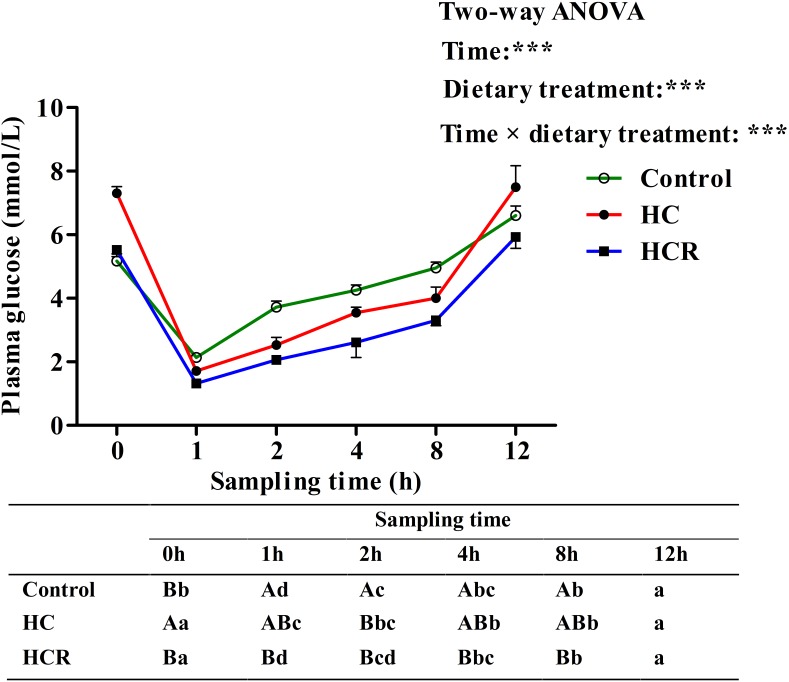
Plasma glucose levels of blunt snout bream subjected to an insulin load after the adaption to different experimental diets. Each data represents the mean of four replicates. Different lower-case letters indicate significant differences (*P* < 0.05) at different time points within each treatment, whereas different capital letters indicate significant differences (*P* < 0.05) among these three treatments at each sampling point. ^∗∗∗^*P* < 0.001. Control, diet with 30% carbohydrate level; HC, diet with 41% carbohydrate level; HCR, diet with 41% carbohydrate and 0.04% resveratrol.

Plasma glucose levels in blunt snout bream after ITT were presented in **Figure 7**. Plasma glucose levels were significantly (*P* < 0.001) affected by sampling time, dietary treatments and their interaction. The insulin administration resulted in a significantly (*P* < 0.05) decreased plasma glucose concentration with lowest levels being obtained at 1 h after injection. Thereafter, plasma glucose levels decreased significantly (*P* < 0.05) to the basal value, which was obtained within 12 h after the insulin load. In terms of dietary treatments, plasma glucose level of the HCR treatment was significantly (*P* < 0.001) lower than that of the other groups. In addition, plasma glucose levels were significantly affected by the interaction between sampling time and dietary treatments with significant (*P* < 0.001) differences observed during the period of 0–8 h among different groups.

## Discussion

In this study, microcrystalline cellulose was incorporated at 13% in diets as a filler to compensate for the carbohydrate levels required. This could not have negative effects on the growth performance of fish, since as a herbivorous fish blunt snout bream has a high tolerance for cellulose than most of the carnivorous and omnivorous species (Li et al., 2013; Zhou C.P. et al., 2013). In fact, in previous studies, 21% cellulose did not result in growth retardation of this species (Zhou C.P. et al., 2013; Li et al., 2014). In the present study, the final weight, feed intake, WGR, SGR, FCR, and PER of fish showed no statistical difference among dietary treatments. This result may be due to the fact that blunt snout bream could utilize higher carbohydrates compared with most carnivorous and omnivorous species (Zhou C. et al., 2013; Li et al., 2014). Hence, high dietary carbohydrate did not result in a severe growth retardation of this species. Unlikely, the NRE, ERE, HSI, and IPF ratio of fish fed the HC diet were significantly higher than those of the control group. The improved NRE and ERE were not surprising since high-carbohydrate diets could depress the gluconeogenic pathway of fish, thus improving energy and protein retention (Sanchez-Muros et al., 1996; Erfanullah, 1998). In addition, high dietary carbohydrates intake could up-regulate the enzymatic activities of GS, FAS, G6PDH and malic enzyme in fish, thus facilitating glycogenesis and adipogenesis (Moreira et al., 2008; Castro et al., 2016). This inevitably led to lipid and glycogen deposition in fish, as might increase body energy contents, thus leading to the increased ERE, HSI, and IPF ratio (Hemre et al., 1995; Erfanullah, 1998). In addition, it is worth noting that resveratrol supplementation resulted in a decrease of these growth parameters (except for FCR) compared to the HC treatment, although no statistical difference was observed. This result was justifiable since resveratrol could depress feed intake in fish, thus leading to a lower growth performance (Zhang et al., 2017; Menoyol et al., 2018). Furthermore, in this study, dietary supplementation of resveratrol remarkably promoted liver glycogen contents of fish compared to the HC group, whereas the opposite was found for both liver and muscle lipid contents. According to a previous study, resveratrol could enhance the glucose-stimulated insulin secretion by improving the functions of pancreatic beta-cells, thus up-regulating GS expression of mammals (Vetterli et al., 2011). In addition, the increased insulin levels usually lead to the dephosphorylation of glycogen phosphorylase (GPase) and glutamine synthetase (GSase) by inhibiting the activities of adenylyl cyclase and decreasing intracellular cyclic adenosine monophosphate (cAMP) formations, thus inhibiting the glycogen breakdown in fish (Moon, 2001). This might inevitably result in a relatively high tissue glycogen contents. As for tissue lipid contents, resveratrol could inhibit fatty acid synthesis through the down-regulation of the transcriptions of ACC, FAS, PPARγ, and SREBP1, all of which are closely involved in hepatic lipogenesis, thus leading to the reduced lipid accumulation (Andrade et al., 2014).

In the present study, hepatic ATP contents and the ATP/AMP ratio were up-regulated in fish fed the HC diet compared to the control group, although no statistical difference was observed. This was reasonable since high-carbohydrate intake usually increases plasma glucose levels, and increases the energy state of cells, thereby resulting in the increased ATP contents coupled with the decreased AMP contents (Magnoni et al., 2012). However, the supplementation of resveratrol induced a remarkable decrease of ATP content and the ATP/AMP ratio compared to the HC group, but the opposite was found for AMP content. According to a previous literature, resveratrol could inhibit the activity of ATP synthase in rats, as might consequently lead to a decrease of ATP content and the ATP/AMP ratio, but the increase of AMP level (Zheng and Ramirez, 2000; Dasgupta and Milbrandt, 2007). In addition, the intake of high dietary carbohydrates also led to a decrease of NAD^+^ contents in liver. This result may be due to the fact that high-carbohydrate intake usually induced a hyperglycemia state of fish, inhibiting the pyruvate dehydrogenase complex, thereby leading to a decreased NAD^+^ content (Belenky et al., 2007; Liu et al., 2013; Xu et al., 2018). Meanwhile, the high value of NAD^+^/NADH ratio was found in the HCR group, which may be due to the fact that resveratrol could increase the phosphorylation of AMPK and promote mitochondrial biogenesis, thus leading to the increased NAD^+^/NADH ratio.

In addition, in this study, fish fed the HC diet attained quietly high plasma levels of glucose, GSP, AGES, insulin and total cholesterol compared to the control group. Generally, high dietary carbohydrate intake usually induces a hyperglycemia state of fish, as might in turn stimulate insulin synthesis and secretion (Polakof et al., 2012; Xu et al., 2017). Excessive glucose reacts non-enzymatically in the blood with the amino groups of plasma proteins to form glycated proteins, and also could enhance the Maillard reaction, thus leading to the increased GSP and AGES levels (Brownlee et al., 1988; Misciagna et al., 2005; Beltramo et al., 2008). The increased total cholesterol level was in line with the common sense, since high-carbohydrate diets could promote the lipogenesis of fish. Furthermore, in this study, resveratrol supplementation led to a significantly decrease of plasma glucose, GSP and total cholesterol concentrations compared to the HC group, while the opposite was found for insulin levels. This might be explained by the following facts that: (1) resveratrol could enhance insulin sensitivity and mitochondrial biogenesis via the activation of both AMPK and SIRT1, thus accelerating glucose uptake in peripheral tissues and consequently lowering plasma glucose and GSP levels (Dasgupta and Milbrandt, 2007; Barger et al., 2008); (2) resveratrol could inhibit lipogenesis in parallel with a down-regulation of lipogenic gene expression, thus lowering plasma total cholesterol levels (Fischer-Posovszky et al., 2010). In addition, the relatively high insulin level observed in the HCR group was also not surprising, due to the fact that resveratrol could activate SIRT1, which in turn regulate the glucose-stimulated insulin secretion in pancreatic β-cells (Vetterli et al., 2011).

Previous studies have shown that both AMPK and SIRT1 play a prominent role in the modulation of cellular energy metabolism (Cantó and Auwerx, 2009; Chau et al., 2010). The activation of AMPK results in an up-regulated cellular NAD^+^ levels, which could in turn activate SIRT1, and subsequently activates PGC-1α through deacetylation. However, such information in fish is still barely available. In this study, hepatic protein contents of t-AMPKα, p-AMPKα, SIRT1, and the p-AMPKα/t-AMPKα ratio as well as transcriptions of AMPKα1, AMPKα2, SIRT1, and PGC-1α were significantly down-regulated by high-carbohydrate feeding, suggesting that a long-term intake of HC diets could induce a poor energy sensing of fish, as might lead to an impaired glucose homeostasis. This result was supported by the fact that AMPK–SIRT1–PGC-1α is an energy sensing network that controls the energy expenditure and metabolic homeostasis of animals (Cantó and Auwerx, 2009). According to previous studies, high dietary carbohydrates intake usually led to the increased intracellular ATP contents and the decreased intracellular AMP contents, thus depressing the activity of AMPKα (Cammisotto et al., 2008; Polakof et al., 2011, 2012). Hence, the decreased activity of SIRT1 and PGC-1α might be a consequence of the decreased AMPKα activity. This was supported by the fact that AMPK could enhance SIRT1 activity by increasing cellular NAD^+^ levels. This might inevitably lead to the deacetylation of PGC-1α, as might consequently stimulate the catabolic pathways and inhibiting the anabolic ones (Cantó et al., 2009; Schirmer et al., 2012). In addition, dietary supplementation of resveratrol significantly up-regulated the protein contents of t-AMPKα, p-AMPKα, and SIRT1, the p-AMPK/t-AMPK ratio as well as the transcriptions of AMPKα1, AMPKα2, SIRT1, and PGC-1α compared with the HC group. These results indicated that a long-term administration of resveratrol could improve the energy sensing of fish fed HC diets. This result might be ascribed to the fact that resveratrol could activate AMPK. This in turn induces the activity of SIRT1, which could activate PGC-1α via deacetylation (Picard et al., 2004).

In the present study, hepatic transcriptions of PEPCK, G6Pase and FBPase were significantly down-regulated in fish fed the HC diet compared to the control group, whereas the opposite was found for that of GLUT 2 and G6PDH. In addition, the transcriptions of both GK and GS were also up-regulated, although no statistical difference was observed. This showed that the long-term intake of a high-carbohydrate diet could inhibit the hepatic gluconeogenesis of blunt snout bream, but promoted glucose transport, glycolysis, glycogenesis, and pentose phosphate pathway. According to previous studies, high-carbohydrate intake usually induced a hyperglycemia state of blunt snout bream (Kamalam et al., 2017; Xu et al., 2017). Excessive glucose was generally taken up by hepatocytes from plasma with the assistance of GLUT2 (Enes et al., 2009). After entering the hepatocytes, overmuch glucose is catabolized via the glycolytic pathway characterized by the up-regulated GK and PK expressions (Enes et al., 2009). Furthermore, hepatic glucose could enhance GS transcription, thus leading to an enhanced glycogenesis (Kamalam et al., 2017). In addition, excessive glucose also inhibited hepatic gluconeogenesis through down-regulating the expressions of PEPCK, G6Pase, and FBPase (Enes et al., 2009; Kamalam et al., 2017), while enhanced the pentose phosphate pathway by up-regulating the transcription of G6PDH (Qiang et al., 2014). Moreover, resveratrol supplementation remarkably increased the transcriptions of GLUT2, and FBPase coupled with a moderate increase of GS expression. This showed that resveratrol could heighten the glucose transport and glycogenesis of fish. According to previous studies, resveratrol treatment could potentiate the glucose-stimulated insulin secretion in pancreatic β-cells, which could in turn promote glucose uptake through irritating GLUT2 in the peripheral tissues, and activate glycogen synthesis by the up-regulation of GS (Sundby et al., 1991; Caruso and Sheridan, 2011; Vetterli et al., 2011). In addition, resveratrol could activate AMPK, as consequently induces the expression of SIRT1, which subsequently activates PGC-1α via deacetylation, thereby leading to the increased FBPase expression (Kim et al., 2013).

In this study, hepatic expressions of SREBP1, FAS, ACCα, and PPARγ were all up-regulated in fish fed the HC diet compared to the control group, whereas the opposite was found for both CPT I and ACO. This indicated that high-carbohydrate intake could promote the fatty acid biosynthesis and fat accumulation of fish, but decrease the fatty acid β-oxidation. This result may be supported by the following facts that: (1) SREBP1, FAS, and ACCα played an important role in hepatic adipogenesis (Qian et al., 2015); (2) PPARγ is considered to play an important role in tissue lipogenesis and lipid deposition (Walczak and Tontonoz, 2002); and (3) both CPTI and ACO are the enzymes closely involved in mitochondrial fatty acid β-oxidation (Lu et al., 2014). Furthermore, it is worth noting that resveratrol supplementation significantly inhibited the transcriptions of SREBP1, FAS, and ACCα, but up-regulated that of CPTI compared to the HC group, indicating that resveratrol could enhance fatty acid β-oxidation and reduce hepatic lipid accumulation of fish. According to a previous study, the up-regulation of SIRT1 by resveratrol triggered the activities of PGC-1α through deacetylation, thereby resulting in a reduced lipogenesis by inhibiting the expressions of SREBP1 and PPARγ in zebrafish (Ran et al., 2017). In fact, it has been shown that resveratrol supplementation could significantly elevate the CPTI and ACO activities in rats, thus preventing liver fat accumulation (Andrade et al., 2014).

After a glucose load, the highest plasma glycemia was obtained at 1 h. The time to reach the highest glucose level in blunt snout bream was similar to that observed in common carp *Cyprinus carpio* and tilapia *Oreochromis niloticus × O. aureus* (Furuichi and Yone, 1981; Lin et al., 1995), but was lower than that of most carnivorous fish like red sea bream *Chrysophrys* major and yellowtail *Seriola quinqueradiata* (Furuichi and Yone, 1981), which required 2–3 h to reach the glucose peak. Furthermore, present data suggested that blunt snout bream require circa 6 h for re-establishing basal values of circulating levels of glucose. This was similar to the results observed in omnivorous tilapia (6 h) (Chen et al., 2017) and common carp (5 h) (Furuichi and Yone, 1981) administrated with the same glucose dose, but was much lower than that of the carnivorous (12–24 h), such as chinook salmon *Oncorhynchus tshawytscha* and European sea bass *Dicentrarchus labrax* (Mazur et al., 1992; Enes et al., 2011). This may be due to the fact that being a herbivorous fish, blunt snout bream might have a higher glucose phosphorylation capacity in muscle and fat tissues (main peripheral tissues) and a stronger inhibition of hepatic gluconeogenesis (Kirchner et al., 2005; Polakof et al., 2012) than most carnivorous ones. In addition, after insulin administration, plasma glucose levels promptly decreased at 1 h, indicating that blunt snout bream could effectively respond to exogenous insulin. The time to reach the glucose lowest in this species was shorter than that of most carnivorous species (Jin et al., 2014, 2017; Deck et al., 2017), which required 3–9 h to attain the minimum value. This was supported by the fact that being a herbivorous freshwater fish, blunt snout bream has a higher capability of glycogen synthesis in peripheral tissue (Polakof et al., 2010) and a stronger inhibition of hepatic gluconeogenesis than most carnivorous species (Navarro et al., 2006). Furthermore, the time hypoglycemia duration in blunt snout bream (within 12 h) was similar to that (within 12 h) of gibel carp *Carassis auratus gibelio* injected the same insulin dose (Jin et al., 2017), but was much faster than that (12–24 h) of carnivorous fish like rainbow trout (Capilla et al., 2002). Furthermore, in terms of dietary treatments, the plasma glucose level of fish fed the HC diet was lower than that of the control group after both the GTT and ITT. This may be due to the fact that fish subjected to high-carbohydrate feeding usually characterize an increased number of insulin receptors, an enhanced capacity for glucose phosphorylation, and an inhibition of hepatic gluconeogenesis (Hemre et al., 2002; Polakof et al., 2012), as might consequently lead to the decreased plasma glucose levels. Furthermore, after glucose and/or insulin load, resveratrol supplementation remarkably decreased plasma glucose levels of fish fed the HC diet, as revealed an improved glucose and insulin tolerance of fish. This result might be due to the following facts that: (1) resveratrol could stimulate glucose uptake by increasing the action of GLUTs in the cytoplasmic membrane, thereby normalizing the blood glucose levels (Vallianou et al., 2013); (2) resveratrol could improve insulin tolerance by the induction of a more efficient insulin signaling via the Akt pathway (Brasnyó et al., 2011), thus resulting in a lower plasma glucose levels.

## Conclusion

In conclusion, the present study suggested that dietary supplementation of resveratrol could improve the energy sensing and glycolipid metabolism of blunt snout bream fed high-carbohydrate diets by activating the AMPK–SIRT1–PGC-1α network, the up-regulation of the genes related to glucose transportation, glycogenesis, and fatty acid β-oxidation coupled with the depression of lipogenesis. In addition, resveratrol supplementation slightly compromised the growth performance of blunt snout bream.

## Author Contributions

W-BL and X-FL designed the experiments. H-JS, CX, B-KW, D-HC, and M-YL carried out the experiments. H-JS, X-FL, CX, B-KW, LZ, and C-YX contributed reagents, materials, and analysis tools. H-JS and X-FL wrote the paper. All authors read and approved the final version of the manuscript.

## Conflict of Interest Statement

The authors declare that the research was conducted in the absence of any commercial or financial relationships that could be construed as a potential conflict of interest.
