# Supplementary material for: Resveratrol Improves the Energy Sensing and Glycolipid Metabolism of Blunt Snout Bream Megalobrama amblycephala Fed High-Carbohydrate Diets by Activating the AMPK–SIRT1–PGC-1α Network
Source: Front Physiol. 2018 Sep 11;9:1258. doi: 10.3389/fphys.2018.01258 (PMC6141669; doi:10.3389/fphys.2018.01258)
Supplement: Supplementary file 1 [file Image_1.pdf]

Supplementary Material

Resveratrol Improves the Energy Sensing and Glycolipid Metabolism of Blunt Snout Bream *Megalobrama amblycephala* Fed High-carbohydrate Diets by Activating the AMPK-SIRT1-PGC-1α Network

Hua-Juan Shi[1], Chao Xu[1], Ming-Yang Liu[2, 3], Bing-Ke Wang[1], Wen-Bin Liu[1], Dan-Hong Chen[1], Li Zhang[1], Chen-Yuan Xu[1], Xiang-Fei Li [1]*

[1] Key Laboratory of Aquaculture Nutrition and Feed Science of Jiangsu Province, College of Animal Science and Technology, Nanjing Agricultural University, No.1 Weigang Road, Nanjing 210095, People's Republic of China.

[2] Wuxi Fisheries College, Nanjing Agricultural University, Wuxi 214081, China.

[3] Key Laboratory of Freshwater Fisheries and Germplasm Resources Utilization, Ministry of Agriculture, Freshwater Fisheries Research Center, Chinese Academy of Fishery Sciences, Wuxi 214081, China.

*Corresponding Author: Key Laboratory of Aquatic Nutrition and Feed Science of Jiangsu Province, College of Animal Science and Technology, Nanjing Agricultural University, No. 1 Weigang Road, Nanjing 210095, Jiangsu Province, People's Republic of China. Tel (Fax): 86-025-84395382. E-mail: xfli@njau.edu.cn (Xiang-Fei Li).

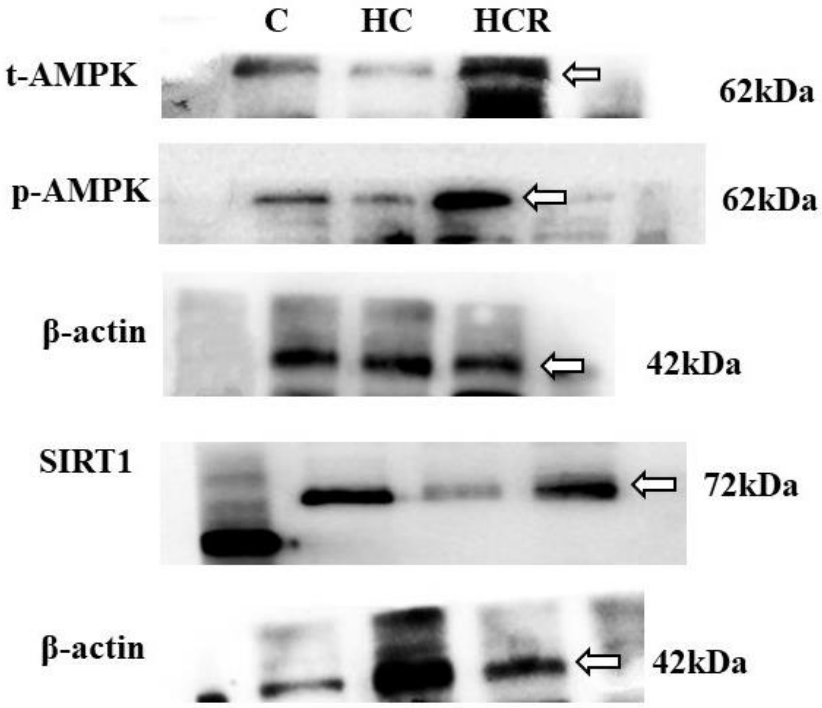

Figure 1. Hepatic t-AMPKα contents, p-AMPKα contents, SIRT1 contents and β-actin levels of blunt snout bream liver fed different experimental diets. Gels were loaded with 20 μg total protein per lane. Control, diet with 30% carbohydrate level; HC, diet with 41% carbohydrate level; HCR, diet with 41% carbohydrate and 0.04% resveratrol. t-AMPKα, AMP-activated protein kinase α; phosphorylated AMP-activated protein kinase α; SIRT1, Sirtuin-1.
